# Bode Phase Angle Signaling of a TB Disease Biomarker

**DOI:** 10.3390/molecules28248100

**Published:** 2023-12-15

**Authors:** Unathi Sidwaba, Kaylin Cleo Januarie, Sixolile Mini, Kefilwe Vanessa Mokwebo, Emmanuel Iwuoha, Usisipho Feleni

**Affiliations:** 1 SensorLab (University of the Western Cape Sensor Laboratories), 4th Floor Chemical Sciences Building, University of the Western Cape, Robert Sobukwe Road, Bellville, Cape Town 7535, South Africa; 2713651@myuwc.ac.za (U.S.); 3241625@myuwc.ac.za (K.C.J.); 3228366@myuwc.ac.za (S.M.); 3696304@myuwc.ac.za (K.V.M.); 2 Institute for Nanotechnology and Water Sustainability (iNanoWS), Florida Campus, College of Science, Engineering and Technology (CSET), University of South Africa (UNISA), Johannesburg 1709, South Africa

**Keywords:** interferon gamma, phase angle, pleural fluid, poly(3,4-propylenedioxythiophene) nanocomposite, tuberculosis diagnosis

## Abstract

Tuberculosis (TB) is a worldwide burden whose total control and eradication remains a challenge due to factors including false positive/negative diagnoses associated with the poor sensitivity of the current diagnostics in immune-compromised and post-vaccinated individuals. As these factors complicate both diagnosis and treatment, the early diagnosis of TB is of pivotal importance towards reaching the universal vision of a TB-free world. Here, an aptasensor for signaling an interferon gamma (IFN-γ) TB biomarker at low levels is reported. The aptasensor was assembled through gold–thiol interactions between poly(3,4-propylenedioxythiophene), gold nanoparticles, and a thiol-modified DNA aptamer specific to IFN-γ. The aptasensor sensitively detected IFN-γ in spiked pleural fluid samples with a detection limit of 0.09 pg/mL within a linear range from 0.2 pg/mL to 1.2 pg/mL. The good performance of the reported aptasensor indicates that it holds the potential for application in the early diagnosis of, in addition to TB, various diseases associated with IFN-γ release in clinical samples.

## 1. Introduction

Tuberculosis (TB) is a highly infectious disease whose severity is aggravated by co-infection with other pathogens as well as communicable and non-communicable diseases such as human immunodeficiency virus (HIV), cryptococcosis, and diabetes mellitus [1,2,3]. Although both TB infection (latent TB infection, LTBI) and TB disease (active TB) can be prevented and cured, enormous factors challenge the measures aiming towards the eradication of the epidemic, as they delay or complicate both diagnosis and treatment. These factors include the dormant behavior of the bacteria which delays symptom presentation, the failure of current diagnostics to solely discriminate LTBI from active TB, the emergence of antibiotic-resistant strains, and, most importantly, the impaired sensitivity and specificity of current diagnostics in children, as well as immune-compromised and post-Bacillus Calmette Guérin (BCG)-vaccinated individuals [4,5,6,7]. As a result, TB-related deaths per annum are estimated at 1.7 million globally [2]. Hence, rapid, and ultrasensitive assays are of pivotal importance to reach the universal vision of a TB-free world, given that the prompt discovery of TB infection will facilitate both preventive and treatment measures in susceptible and infected individuals.

Amongst the measures addressing the specificity and sensitivity issues associated with current TB diagnostics, aptamers and conducting polymer-based immobilization films employing nanoscale materials have gained attention as analyte-specific and signal-boosting agents in electrochemical assays, respectively. Such a combination yields highly selective and sensitive point-of-care electrochemical assays with additional features such as low detection limits, portability, easy handling, and faster response times [8,9,10]. Currently, conducting polymer nanocomposites are among the most widely employed immobilization materials during biosensor fabrication as they combine the synergistic properties of individual components. These nanocomposites are not only biocompatible for binding and retaining the activity of biomolecules in biosensors but they also determine the performance of the biosensors and exhibit fantastic functionalities for catalysis, as well as medical, optical, and energy storage applications [11,12]. Poly(3,4-propylenedioxythiophene) (PProDOT) is a thiophene-derivatized polymer whose functionality has been manipulated for application in electrochromic devices, supercapacitors, and eyewear due to its excellent capacitive and electrochromic behavior [13,14,15]. Contrary to its analogue, poly(3,4-ethylenedioxythiophene) (PEDOT), PProDOT has not been widely applied in electrochemical biosensors, although its potential for being applied in such sensors has been documented by different researchers [16,17].

Intensive work has been conducted on TB diagnosis through the signaling of its immune response markers (IFN-γ, lipoarabinomannan (LAM), early secreted antigenic target 6 (ESAT-6)), *mycobacterium tuberculosis (MTb)* DNA strains, and the bacteria in *MTb* cells in different matrices such as serum, blood, urine, pleural fluid, and sputum [18,19,20,21,22,23,24]. Amongst the TB biomarkers mentioned above, IFN-γ is the most versatile, as its various levels are associated with specific diseases and stages of disease progression where low levels (≤15 picogram per milliliter) are associated with TB, HIV, and leukemia, while higher levels (>1 nanogram per milliliter) are associated with Johne’s disease [25,26].

Herein, this study reports an electrochemical aptasensor system for early TB diagnosis based on the low-level signaling of IFN-γ in pleural fluid, monitored through phase angle maxima. The sensor system consists of PProDOT, which was employed as both a redox probe and a receiving matrix and whose capability was enhanced through the incorporation of gold nanoparticles (AuNPs). The PProDOT–AuNP nanocomposite is a layer-by-layer deposited film with the aim of maximizing the surface area and exposing a great yield of AuNPs for capturing the thiol-modified aptamer specific to IFN-γ. After the deposition of the thiolated aptamer and the blocking of non-specific adsorptions through the gold surface, the fabricated aptasensor system detected IFN-γ with a detection limit of 0.11 pg/mL and a linear range extending from 0.2 pg/mL to 1.2 pg/mL in spiked pleural fluid. The aptasensor’s performance was compared to other IFN-γ detection sensors, and its performance indicates that it has the potential for application in the early diagnosis of TB in clinical samples.

## 2. Results and Discussion

### The Design and Fabrication of the Aptasensor

**Figure 1:**
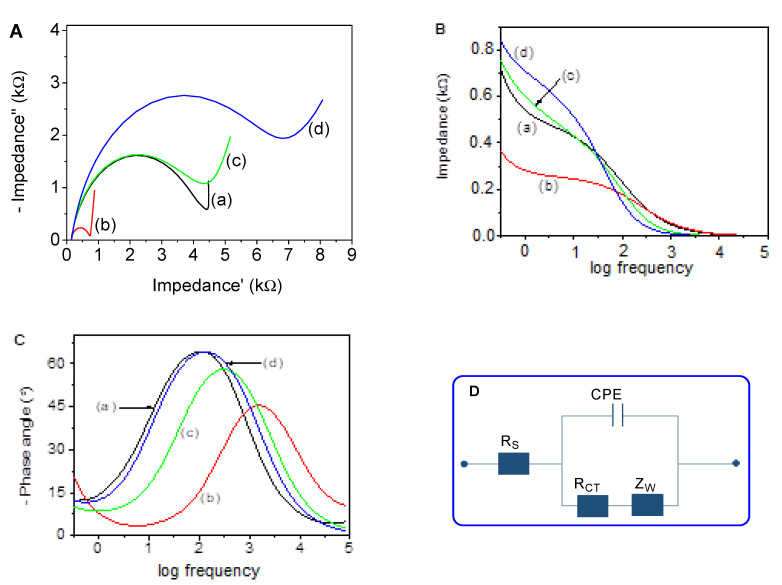
EIS Nyquist (**A**) and bode plots (**B**,**C**) of (a) the PProDOT, (b) PProDOT–AuNPs, (c) PProDOT–AuNPs-Aptamer, and (d) PProDOT–AuNPs–Aptamer–MCH (aptasensor) in the frequency range from 0.1 Hz to 100 kHz with 10 mV amplitude in PBS, pH 7.4. (**D**) The Randles equivalent circuit was used to fit experimental data.

The feasibility of using the PProDOT nanocomposite film as a redox probe was investigated using EIS in phosphate buffer, pH 7.4. At high frequency, the Nyquist plot (Figure 1A) shows an equivalent series resistance of 0.15 kΩ, and this combines the internal resistance of the active material (GCE and PProDOT) and solution resistance. At mid/high frequencies, a semicircle whose diameter is directly proportional to the electrochemical charge transfer resistance (R_CT_) at the electrode/electrolyte interface is observed. The recorded R_Ct_ value is 4.8 kΩ. At low frequencies, a tail indicative of the diffusion of anions to the electrode surface is observed [27]. Furthermore, the tail runs parallel to the imaginary axis with a corresponding total impedance of 0.71 kΩ and a maximum phase angle of 64.2° (observed from the bode plots in Figure 1B,C). Assembling a layer of AuNPs onto the PProDOT film enhances charge transfer [25]; hence, a decrease in R_ot_ (reduced semicircle diameter) and a decrease in total impedance to 0.8 kΩ and 0.37 kΩ, respectively, are observed. The presence of AuNPs endows the film with a pseudo-capacitive behavior (exhibited by a phase angle deviation of 45.4° to the imaginary axis). The enhanced performance of the PProDOT–AuNP film can be attributed to the AuNPs’ high surface area and the protonation of the PProDOT to a state that electrostatically interacts with the phosphate anions in the electrolyte, thus promoting electron transfer and decreasing resistance [28]. This makes the film a suitable redox probe for monitoring the phase angle changes that will be induced by the different components of the aptasensor. The equivalent circuit (Figure 1D) used for the analysis of these fabrication steps represents the charge transfer resistance as R_CT_, the capacitive behavior of the functionalized electrode–electrolyte interface as the constant phase element (CPE), the internal resistance of the electrode combined with that of the electrolyte as R_S_, and the diffusion process as the Warburg element represented by Z_W_ [29].

Upon the fabrication of the aptasensor, the incubation of the PProDOT–AuNPs film with the thiol-modified anti-IFN-γ aptamer induces a repulsion between the negatively charged moieties of the aptamer and the electrolyte anions [30]. With specific reference to the nature of the fabrication process entailed here, the aptamer binds to the AuNPs and forms a layer which, due to the steric hindrance effect of the aptamer, increases the thickness at the interface and therefore the distance for electron exchange between the modified GCE and the electrolyte anions. This incubation contributes to a retarded charge transfer with a corresponding R_CT_ of 4.9 kΩ, a total impedance of 0.75 kΩ, and a maximum phase angle of 58.1°. The subsequent blocking of the unbound surface with the MCH backfiller further increases the charge transfer resistance to 7.4 kΩ, accompanied by an impedance of 0.84 kΩ and a maximum phase angle of 64.1°. Based on these results, one can determine whether the aptasensor interface based on PProDOT has been successfully fabricated according to Figure 1.

#### Bode Phase Angle Detection of IFN-γ

**Figure 2:**
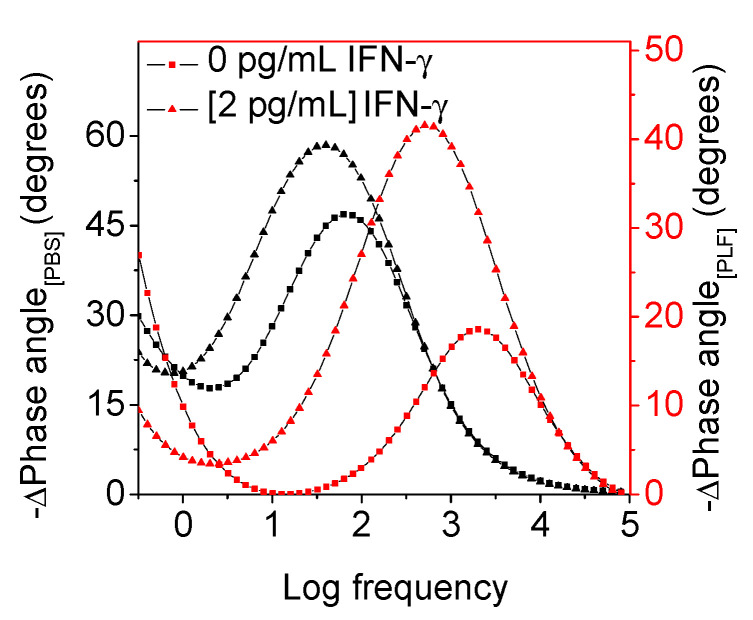
Phase angle signal in the absence (0 pg/mL) and presence (2 pg/mL) of IFN-γ upon incubation in phosphate buffer (left axis) and pleural fluid (right axis). The higher signal change in PLF is associated with the complexity of PLF.

The response of the developed aptasensor was tested in the absence and presence of IFN-γ at a concentration of 2 pg/mL in phosphate buffer and spiked pleural fluid. As shown Figure 2, incubating the aptasensor with IFN-γ in PBS showed a phase angle difference of 11° and a frequency separation of 23 Hz. This difference is determined from the signal observed at 46.9° and 63 Hz before incubation and at 58° and 40 Hz in the presence of IFN-γ. A similar trend was exhibited after incubating aptasensor with IFN-γ in spiked pleural fluid. The phase angle shifted to 42° at 500 Hz, compared to the 19° observed at 1000 Hz in the absence of IFN-γ. However, this behavior constitutes a phase angle difference of 23° and a frequency separation of 500 Hz, which are both two-fold magnitudes compared to the performance of the aptasensor in PBS. Under both environments, PBS and PLF, the increase in the phase angle determined by the increase in R_CT_ are attributed to the aptasensor–IFN-γ complex, which further increases the distance for electron exchange; hence, the retarded diffusion of anions (elaborated by the shift to lower frequencies) is observed [31].

**Figure 3:**
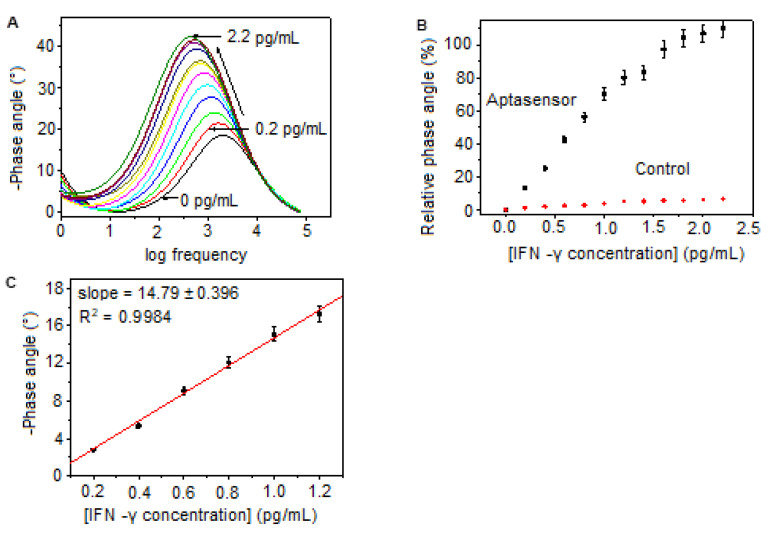
(**A**) Aptasensor responses of IFN-γ ranging from 0.2 pg/mL to 2.2 pg/mL in spiked PLF between 0.1 Hz and 100 kHz at an amplitude of 10 mV. (**B**) The calibration curve for the aptasensor during the detection of IFN-γ in comparison to the control sensor fabricated without a recognition element. (**C**). Linear curve drawn from the linear regression of (**A**), from which a detection limit of 0.09 pg/mL was determined. The error bars represent the averages of three experimental results.

The applicability of the developed aptasensor was investigated in the presence of IFN-γ in spiked pleural fluid at low-level concentrations ranging from 0 pg/mL to 2.2 pg/mL (Figure 3A). Incubating the aptasensor with increasing concentrations of IFN-γ shows an increase in phase angle maxima, with corresponding frequencies shifting towards lower values as a result of the aptasensor–IFN-γ complex, which changes the capacitive behavior at the electrode–electrolyte interface, while the increasing amount of IFN-γ increases the repulsion of the electrolyte anions, thus retarding the electron exchange between the modified electrode and the electrolyte [32]. The aptasensor system was validated against a control sensor fabricated under the same conditions (excluding the aptamer). Due to the absence of recognition, binding, and the formation of an aptasensor–IFN-γ complex, the control sensor only shows a slight change in relative phase angle response, while the response of the aptasensor increases exponentially with increasing doses of IFN-γ (Figure 3B). The aptasensor’s dose response curve exhibits a linear dependence of the relative phase angle signal on IFN-γ concentration (Figure 3C), with an excellent correlation (R^2^ = 0.9984) from 0.2 pg/mL to 1.2 pg/mL, from which a detection limit of 0.11 pg/mL was determined. This detection limit is comparable with studies based on IFN-γ quantification in different samples. Regarding the early diagnosis of TB, Abnous Khalil and his group fabricated an aptasensor based on a triple helix molecular structure and methylene blue redox probe [33]. Their aptasensor sensitively quantified IFN-γ in spiked serum samples with a detection limit of 3 pg/mL using square wave voltammetry oxidation current signals. In a different study, Wang and his research group developed an immunosensor for IFN-γ detection in serum and obtained a detection limit of 0.12 pg/mL [34]. The performance of the aptasensor developed in this study highlights its potential for application in the quantification of IFN-γ at very low levels in clinical samples for the prompt diagnosis of TB and other diseases associated with IFN-γ release.

## 3. Materials and Methods

### 3.1. Materials

Chloroauric chloride (HAuCl_4_), 3,4-propylenedioxythiophene (ProDOT), sodium dodecyl sulphate (SDS), 6-mercapto-1-hexanol (MCH), hydrochloric acid (HCl), sodium phosphates (NaH_2_PO_4_·2H_2_O and Na_2_HPO_4_·2H_2_O), acetonitrile, and trisodium citrate were purchased from Sigma-Aldrich (Kempton park, South Africa). All solutions were prepared using deionized water purified through the Milli-Q Plus system (18.2 MΩ·cm). The tuberculosis (TB) biomarker (interferon gamma (IFN-γ)), the anti-IFN-γ single-stranded DNA (*ss*DNA) aptamer modified with a thiol group at the 5′ end, and pleural fluid were obtained from the Lung Infection and Immunity Unit (School of Medicine, University of Cape Town, South Africa). The 29-mer anti-IFN-γ aptamer had the following sequence: 5′-5thiolMCS-D/CGG CGA AGG CAC GTG TGG GGT GGT CGC GT-3′.

### 3.2. Apparatus 

All experiments were conducted using a CHI760E electrochemical workstation and a conventional three-electrode system with a glassy carbon working electrode (GCE, 0.071 cm^2^), a platinum wire counter electrode, and a silver chloride (Ag/AgCl, 3 M NaCl) reference electrode. Electrochemical impedance spectra (EIS) were acquired in a frequency range spanning from 0.3 Hz to 100 kHz at 10 points per decade and at an excitation potential of 10 mV. 

### 3.3. Synthesis of PProDOT

An acetonitrile solution of ProDOT was diluted to 26.7 mg/mL using 0.1 M hydrochloric acid (HCl) and 0.1 M SDS under constant stirring at room temperature. After the rapid addition of 15 mM chloroauric chloride, the solution immediately turned green, with visible precipitates indicative of polymer deposition. After 4 h, the polymer was purified in acetonitrile and was collected as a greenish-blue powder.

### 3.4. Assembly of the Aptasensor and Detection of IFN-γ

**Scheme 1:**
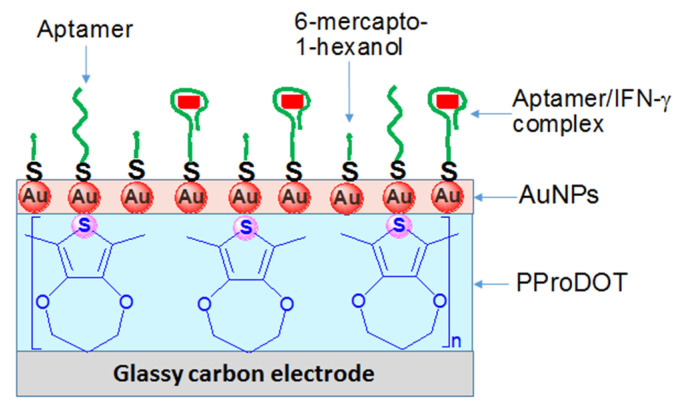
Illustration of the aptasensor fabrication strategy based on the gold–thiol interactions between the PProDOT, AuNPs, and the thiol-modified anti-IFN-γ aptamer. To avoid non-specific adsorption between the analyte, 6-mercapto-1-hexanol (MCH) backfills the free PProDOT–AuNP film. When introduced, IFN-γ forms a complex with the aptamer.

A PProDOT suspension (6 µL) was drop coated onto a glassy carbon electrode, followed by the dropcoating of a layer of gold nanoparticles (AuNPs, 5 µL, prepared according to the procedure in [27]), yielding a layer-by-layer deposited PProDOT-AuNP nanocomposite film. The aptasensor was fabricated by dropcoating the ssDNA aptamer (5 µM) onto the PProDOT/AuNP nanocomposite film, followed by the addition of 6-mercapto-1-hexnol (MCH) to avoid and minimize non-specific adsorption between IFN-γ and the AuNPs. The anti-IFN-γ aptamer in binding buffer (containing 0.1 M phosphate buffer, 10 mM KCl and 2 mM MgCl_2_) was pre-treated by heating at 90 °C for 10 min followed by rapid cooling at 4 °C for 5 min and cooling at room temperature for 10 min. Then, various IFN-γ concentrations from 0.2 pg/mL to 2.2 pg/mL were electrochemically detected in spiked pleural fluid by monitoring induced phase angle changes as a function of frequency. Please see Scheme 1 below.

## 4. Conclusions and Future Recommendations

This study reports, for the first time, an electrochemical aptasensor based on poly(3,4-propylenedioxythiophene) (PProDOT). Through the amplification effect of the gold nanoparticles electrostatically attached to the PProDOT through the gold–thiol interactions, the resulting film demonstrated excellent electrical conductivity, which made the film a suitable candidate for signaling-induced signal changes during the fabrication and application of the proposed aptasensor. The fabrication of the aptasensor was monitored and confirmed through bode phase angle signal changes (at corresponding frequencies) induced by the different components of the aptasensor.

During application, the aptasensor showed good performance, as validated via a comparison with the control sensor, which lacks the IFN-γ-recognizing element, the aptamer. The practical application of this aptasensor was realized in spiked pleural fluid samples, and a relatively low detection limit of 0.09 pg/mL and a sensitivity of 14.79 ± 0.396°·(pg/mL) were achieved. The aptasensor was constructed with a specific and highly selective thiolated aptamer which was shown in a previous work (Januarie et al., 2023) to not exhibit signals in the presence of other proteins (i.e., interfering species), such as interleukin-2 (IL-2, 120 pg/mL), immunoglobulin G (IgG, 120 pg/mL), human serum albumin (HSA, 120 pg/mL), bovine serum albumin (BSA, 120 pg/mL), and interferon gamma (10 pg/mL) [35]. Due to the association of IFN-γ levels with diverse diseases and their progression stages, this aptasensor could be used for the early detection of diseases linked to IFN-γ release. Also, by simply changing the aptamer, various disease biomarkers can be detected through the bode phase angle signals using the PProDOT platform as the redox probe and immobilization host. Interestingly, the aptasensor system demonstrated in this study will have future applications in infectious disease monitoring, immunology, and cancer research. However, to realize out-of-laboratory practical application by clinicians, medical practitioners, and all other individuals, this aptasensor system would need to be integrated into an automated, portable, and user-friendly device.

## Data Availability

The data presented in this study are available from the corresponding author upon request.
